# Tracking animal movements using biomarkers in tail hairs: a novel approach for animal geolocating from sulfur isoscapes

**DOI:** 10.1186/s40462-020-00222-w

**Published:** 2020-09-18

**Authors:** Zabibu Kabalika, Thomas A. Morrison, Rona A. R. McGill, Linus K. Munishi, Divine Ekwem, Wilson Leonidas Mahene, Alex L. Lobora, Jason Newton, Juan M. Morales, Daniel T. Haydon, Grant G. J. C. Hopcraft

**Affiliations:** 1 Institute of Biodiversity, Animal Health and Comparative Medicine, University of Glasgow, Graham Kerr building, Glasgow, G12 8QQ UK; 2 National Environmental Isotope Facility, Scottish Universities Environmental Research Centre, University of Glasgow, Glasgow, G75 0QF UK; 3 Nelson Mandela African Institution of Science and Technology (NM-AIST), P.O. Box 447, Arusha, Tanzania; 4 Tanzania Wildlife Research Institute (TAWIRI), P.O Box 661, Arusha, Tanzania; 5 INIBIOMA, CONICET, Universidad Nacional del Comahue, San Carlos de Bariloche, Argentina

## Abstract

**Background:**

Current animal tracking studies are most often based on the application of external geolocators such as GPS and radio transmitters. While these technologies provide detailed movement data, they are costly to acquire and maintain, which often restricts sample sizes. Furthermore, deploying external geolocators requires physically capturing and recapturing of animals, which poses an additional welfare concern. Natural biomarkers provide an alternative, non-invasive approach for addressing a range of geolocation questions and can, because of relatively low cost, be collected from many individuals thereby broadening the scope for population-wide inference.

**Methods:**

We developed a low-cost, minimally invasive method for distinguishing between local versus non-local movements of cattle using sulfur isotope ratios (δ^34^S) in cattle tail hair collected in the Greater Serengeti Ecosystem, Tanzania.

**Results:**

We used a Generalized Additive Model to generate a predicted δ^34^S isoscape across the study area. This isoscape was constructed using spatial smoothers and underpinned by the positive relationship between δ^34^S values and lithology. We then established a strong relationship between δ^34^S from recent sections of cattle tail hair and the δ^34^S from grasses sampled in the immediate vicinity of an individual’s location, suggesting δ^34^S in the hair reflects the δ^34^S in the environment. By combining uncertainty in estimation of the isoscape, with predictions of tail hair δ^34^S given an animal’s position in the isoscape we estimated the anisotropic distribution of travel distances across the Serengeti ecosystem sufficient to detect movement using sulfur stable isotopes.

**Conclusions:**

While the focus of our study was on cattle, this approach can be modified to understand movements in other mobile organisms where the sulfur isoscape is sufficiently heterogeneous relative to the spatial scale of animal movements and where tracking with traditional methods is difficult.

## Background

Movement is a fundamental characteristic of life [1], yet its quantification across individuals and populations has remained a major methodological challenge in the field of movement ecology. Common animal tracking techniques such as GPS and radio transmitters [2] are time-intensive to collect, require expensive equipment and pose welfare concerns [3], and therefore may not be available in all settings nor necessary for addressing certain questions. For instance, characterizing population-level variation in movement patterns, such as the proportion of residents versus migrants, is likely to be inaccurate if one can only tag a handful of individuals over a relatively short period of time [4]. Forensically recreating movement paths from dead animals, or studying historical connectivity from archived specimens, or studying landscape connectivity, can provide useful insights into the drivers of population dynamics. There are few techniques currently available for retrospective animal movement tracking that allow the characterization of population-wide movements. The use of intrinsic markers to infer location of animals [5] offers a relatively low-cost, noninvasive solution to study animal movement patterns.

Intrinsic markers are natural biological or biogeochemical tags that can be retrieved from animals’ tissues [5]. Biogeochemical markers are particularly promising for studying movements because they form links between seasons and across populations, and they give time-integrated information which can directly be linked to geographical regions [5]. Stable isotopes of key elements, particularly hydrogen (δ^2^H) and sulfur (δ^34^S), are popular geolocators [5]. Hydrogen isotopes are used to make inferences of long-distance migration because they vary over latitudinal and continental gradients, reflecting local precipitation patterns [6]. Sulfur isotopes have been popular in movement studies such as tracking distance to the sea [7] and in dietary studies for making inferences about marine and marsh food webs [8]. Sulfur isotope fractionation between soil and plants [9–11] is around − 2‰ and from − 1‰ to + 2‰ between animal diet and different tissues [12–16], suggesting that sulfur is a useful geolocator in animal tissue because it is largely reflective of δ^34^S of the local geology [12, 17]. There are only two published values of the offset between diet and mammalian keratin: − 1‰ [12] and 1.2 ± 0.3 ‰ [18], with the latter study suggesting that offsets are higher for hair than other tissues. The combination of inert biological material that acts as a natural biologger for geolocating animals and continuously growing tissue that does not erode easily, can provide unique time series information about animal movement. This is because as individual animals move across distinct soils or between food webs, tissues that grow continuously (e.g. hair) retain the isotopic signatures of their present and previous feeding locations [17], potentially enabling ecologists to infer movement patterns from them.

This study demonstrates how variation in δ^34^*S* along cattle tail hair can be used to study animal movement retrospectively. We first assess the variation of δ^34^S across environmental space in the Serengeti landscape, and develop a sulfur isoscape for the ecosystem. We test the hypothesis that variation in δ^34^S in grass samples is reflected in sections of the most recent growth of tail hair, which indicates whether tail hair has the potential to be used as a natural bio-logger of geolocation in cattle. Lastly, we establish the distribution of travel distances across the Serengeti ecosystem sufficient to detect movement using sulfur stable isotopes. Agropastoral cattle provide an ideal system for developing these methods because 1) these animals often move long distances; 2) their movements play important roles in human-wildlife conflict and the epidemiology of livestock diseases; 3) cattle owners can help verify the animal’s movement history; and 4) cattle are easier and cheaper to capture and sample than wild animals.

## Materials and methods

### Study area

**Fig. 1:**
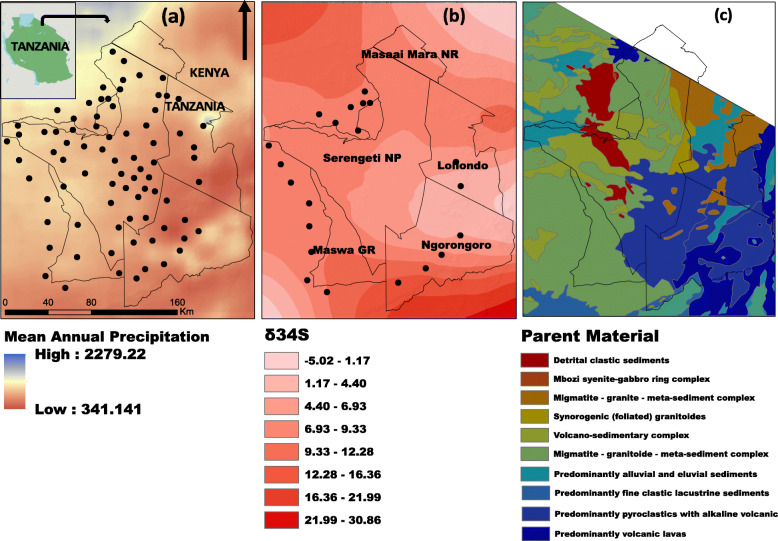
**a** The mean annual precipitation (**b**) the interpolated sulfur isoscape based on the output of a spatial GAM and (**c**) the underlying geology of the area. The boundaries of protected areas in the Serengeti ecosystem are illustrated with black lines. Sampling locations for (**a**) grass and (**b**) cattle are illustrated with black points

The study was conducted in the Serengeti ecosystem, Tanzania, (Fig. 1a), a landscape that is approximately 25,000 km^2^ in area [19, 20] and located between 34^o^ and 36^o^ E, and 1^o^ and 3^o^ N [21]. The ecosystem is characterized by a subtropical climate, with a dry and relatively cool season from late May to August, and a warmer dry season from September to October. Rainfall is highly variable but normally peaks in December, and between March and May [21, 22]. The area’s savanna vegetation is strongly influenced by soil type and rainfall [22] (Fig. 1a). The ecosystem is home to a diverse assemblage of both wild and domestic ungulates, including the largest terrestrial mammal migration in the world [23]. The soils underlying the ecosystem are highly heterogeneous and largely volcanic in nature [24]. The Eastern portion of the ecosystem, including the Serengeti plains, comprises alkaline soils derived from tephra deposited more than twenty thousand years ago from Ngorongoro rift region and more recently from the Oldonyo Lengai volcano [25], the world’s only active carbonatite volcano [26, 27]. The Western portions of the ecosystem are dominated by older alluvial soils, formed from the erosion of Precambrian volcanic rocks and banded ironstones [25, 27, 28]. Other parts of the ecosystem including north of the Mara river and crater highlands are dominated by heavily leached soils from older parent materials including complex granite, and volcanic rocks [25, 29, 30] (Fig. 1c). In addition to a number of protected areas, such as Serengeti National Park in Tanzania and the Masai Mara National Reserve in Kenya [31], the ecosystem supports a large human population consisting of pastoralists and agro-pastoralists living in close proximity to protected areas boundaries.

### Data collection

All data collection including questionnaires, hair and grass samples was conducted from July 2017 to April 2018.

#### Environmental sampling

We collected grass samples from across the ecosystem to establish the spatial pattern of sulfur in the landscape. Grass samples were collected from 20 randomly selected villages bordering the Serengeti National Park but still within the Serengeti ecosystem (Fig. 1a). A total of 116 grass samples were collected and analyzed for δ^34^S to create a landscape-level isoscape. To collect grass samples, a 4 × 4 m plot was laid out and five sub samples (one from each corner and one at the middle) in 25 × 25 cm quadrats were clipped to ground level and pooled together to make one single sample from each site [32]. In addition, grass samples were collected (using the same sampling method) from each village where cattle were sampled to compare local versus landscape level variation. At each village, grass samples were collected from three random points located in non-cultivated grazing fields and at least 100 m away from roads and rivers. This minimized any potential sulfur contamination from industrial fertilizers, vehicle exhaust and road dust. Collected grass samples were kept in a paper envelope and stored in an open area at room temperature to prevent microbial activities and fungal development.

#### Cattle hair sampling

In each of the 20 villages where grass samples were collected, between one and three cattle from random households were sampled for tail hairs, making a total of 46 tail hair samples. Tail hair samples were obtained by pulling hairs from the base of the tail of each animal. Pulling helped to remove the entire hair root, which represents the most recent feeding history of an animal. Cattle age, sex and color were recorded during sampling. After hair collection, all hairs from an individual were aligned by root to standardize time zero (i.e. the most recent time), tied together and stored at room temperature [33, 34].

#### Questionnaire

A set of eight close-ended questions (See Additional information 1: Appendix 1) focused on exploring the movement history of cattle was posed to each cattle owner in relation to the sampled cattle. Questionnaires to cattle owners provided supplementary information on whether cattle had moved beyond the grazing area of the village or had been recently purchased from a neighboring village during the 5 months period prior to hair sampling. In addition to this binary response variable (moved versus not moved), these questionnaires also provided an estimate of distance travelled based on descriptions of the villages from which cattle had reportedly been moved.

### Sample preparation for stable isotope analysis

For grass samples, all non-grass species and debris were removed from samples prior to analysis. Grasses were thoroughly washed in double distilled water (DDS) to remove any soil. Grass samples were oven dried at 60 °C for 48 h, pulverized into a fine powder and weighed (6.1–6.5 mg) into tin capsules [32], ready for isotopic analysis.

The range of hair bundles’ length varied between 10 and 25 cm for adult cattle and between 5 and 7 cm for calves. The growth rate of cattle tail hair has been estimated to be 0.76 mm per day [35]. In this study, tail hairs were sectioned into 10 × 8 mm segments representing 105 days of growth for adult cattle and 5–9 × 8 mm segments representing 52 to 95 days for calves. All samples were thoroughly washed in 2:1 chloroform:methanol to remove the impurities [36], and then rinsed with DDS to remove the remnants of solvent. This process was repeated twice to ensure all possible contaminants had been removed from the samples. Samples were then oven dried, ground to powder and weighed (1.0–1.3 mg) into tin capsules as described above.

### Stable isotope ratio analysis

All laboratory analyses for stable isotope ratios were performed at the NERC Life Sciences Mass Spectrometry Facility hosted by the Scottish Universities Environmental Research Centre (SUERC). All sample analyses were undertaken using a Pyrocube elemental analyser (Elementar nalysensysteme, Langenselbold, Germany) coupled to a VisION isotope ratio mass spectrometer (Elementar UK, Cheadle Hulme, Stockport, UK). Laboratory standards methanesulfonamide/Gelatine (MSAG2), methionine/alanine/glycine/gelatine (M2) and sulfanilamide/alanine/gelatine (SAAG2) were repeated after every 10 samples and were used to correct for linearity and instrument drift over a 72-h analytical run. The isotope ratios for lab standards are determined relative to a range of international standards from IAEA (Vienna, Austria) and USGS (Reston, VA, USA). The analytical precision for sulfur isotopes was better than 0.7‰. The isotope ratios are expressed in the delta (δ) notation in parts per million (‰): δX = [(Rsample/Rstandard)-1] where X = ^34^S and R = the ratio of ^34^S/^32^S isotopes in a given sample compared with V- CDT (Vienna - Canyon Diablo Troilite).

### Sulfur isoscape

To develop a sulfur isoscape for Serengeti, we fitted a spatial Generalized Additive Model (GAM) using the *mgcv* package [37, 38]. The fitted GAM of sulfur isotope ratios was modeled as a function of spatial and environmental variables including mean annual precipitation (MAP), Normalized Difference Vegetation Index (NDVI), soil exchangeable bases (CEC), the underlying geology (geology layer), elevation and the longitude and latitude. Longitude and latitude were modelled as a tensor product smoother to allow for construction of a single model matrix with multiple penalties [38]. All environmental variables were prepared in R [39] using the *sp, raster, rdgal* and *rgeos* packages [40–44]. To characterize mean annual rainfall (MAP), we averaged African monthly data from The Climate Hazards Group Infrared Precipitation with station data (CHIRPS) 1981 and 2018 [45, 46]. CHIRPS integrates 0.05° resolution satellite imagery with in-situ station data to create gridded rainfall time series for trend analysis and seasonal drought monitoring [45]. The underlying geology and parent material for different soil types across the Serengeti ecosystem were characterized from the Minerogenic Map of Tanzania layer, from Geological Survey of Tanzania [30]. The underlying geology was classified using lithology (Fig. 1c) of the parent material. Model selection was based on Akaike Information Criterion (AIC, weight = 0.99) (See Additional file 1: Appendix 2) from the *stats* package [47]. Our final model included lithology as the main environmental predictor and longitude and latitude as a tensor product smoother. The model was analyzed for goodness of fit with the *gam.check* function from *mgcv* package [38] and was subsequently used to predict sulfur isotope values (*δ*34*S*_*L*, *j*_) across the entire ecosystem (i.e. the ‘isoscape’) together with the standard deviation that captured the prediction uncertainty (*σ*_*L*_). The predicted δ^34^S isoscape was at the same spatial resolution as the geology layer (5 km^2^ per pixel).

### Validation of δ^34^S methodology

To understand whether δ^34^S in the i^th^ tail hair sample from the j^th^ individual (δ^34^S _*T,i,j*_) linearly reflects the local δ^34^S signature in vegetation (δ^34^S_*L,j*_), and to account for multiple observations of tail hair from the same individual, and that the true values of explanatory variable (*M*_*L,j*_) are latent, and only observed with error (*σ*_*M*_) we constructed a latent ‘error in variables’ model wherein:

$$ {\displaystyle \begin{array}{c}\delta 34{S}_{T,i,j}\sim {\alpha}_j+\beta {M}_{L,j}+{\epsilon}_i\\ {}\delta 34{S}_{L,j}\sim N\left({M}_{L,j},{\sigma}_M\right)\end{array}} $$

The model was fitted in Stan (Stan version 2.23, [48] using the R interface RStan version 2.19), using 3 chains for 10,000 iterations after 5000 as warmup, thinning to generate 3000 posterior samples per parameter. We used weakly informative priors [49] for all parameters: for regression coefficients we used *t* distributions with 3 degrees of freedom and for standard deviations half t with 3 degrees of freedom, except for *σ*_*M*_ where the prior was modelled as *N*(*σ*_*L*_, 0.25). We used the fitted model to generate predictions and associated uncertainty in these predictions for each observation from the most recent segment of tail hair.

### Studying movements using δ34S across the Serengeti sulfur isoscape

Relocation data from 49 GPS collared cattle in Western Serengeti (Ekwem, 2020 [50];) suggested that cattle rarely moved farther than 5 km from their home bomas (i.e. only 1.7% of relocations). Therefore, we expected longer distance movement to be relatively rare. To determine how far an animal would need to travel in order to robustly detect movement from δ^34^S tail hair signatures, we identified 50,000 random pairs of points across the isoscape, predicted δ^34^S_*L*_ for each point, and estimated the mean distance an animal needed to move in order to detect statistically significant differences in isotope values in its tail hair, given the propagated uncertainty in predicting values of δ^34^S_*L*_ from the landscape data, and δ^34^S_*T*_ from δ^34^S_*L*_. We then compared the output with our actual distances travelled by cattle established from the questionnaire (above).

## Results

### Variation of sulfur stable isotope ratios across the Serengeti ecosystem

δ^34^S values of grass range between + 2.82 ‰ and + 13.04 ‰ (See Additional file 1: Appendix 3), consistent with the terrestrial nature of the ecosystem [51]. Our final predicted model of δ^34^S values in Serengeti included lithology as the main environmental predictor, and the latitudes and longitudes and their interaction as spatial smoothing parameters (AIC weight = 0.99: See Additional file 1: Appendix 2). From the GAM, we identified the following statistically significant relationships; a) a positive relationship between sulfur isotope ratios and pyroclastic-alkaline volcanic lavas (β = 0.079 ± 0.029, t = 2.699, *p* = 0.008) and b) a negative relationship between sulfur isotope ratios and volcanic ash/tephra (β = -0.05 ± 0.023, t = 2.175, *p* = 0.032). Other relationships that were not statistically significant from the model included: a negative relationship between sulfur isotopes ratios and granitoids (β = -0.021 ± 0.015, t = 1.345, *p* = 0.181) and between sulfur isotope ratios and volcanic lava (β = -0.016 ± 0.017, t = 0.975, *p* = 0.332), as well as a positive relationship between sulfur isotope ratios and mafic volcanic meta-basalts (β = 0.032 ± 0.021, t = 1.533, *p* = 0.128). The mean standard deviation of predictions estimated directly from the GAM was *σ*_*L*_ = 1.00, and inferred from the full model, *σ*_*M*_ = 1.810.

### Relationship between cattle locations and tail hair isotope values

**Fig. 2:**
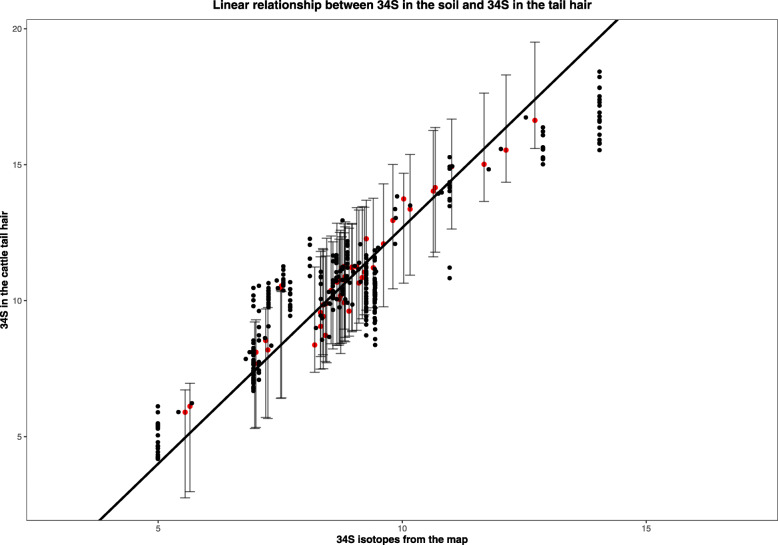
The observed relationship between δ^34^S in grass and the most recent segment of the cattle tail hair, suggesting that δ^34^S in the hair reflects the δ^34^S in the landscape and can be used as a reliable biomarker of location. Red points (slightly ‘jittered’ for clearer visualization), show the most recent segment of a tail hair (i.e. the root), and black points show the rest of the segments in the tail hair

The ‘error in variables’ model showed good convergence and effective sample sizes for all posteriors (Rhat < 1.01, neff > 1400). The δ^34^S values of grasses from locations where tail hair was sampled, and the most recent tail hair section were strongly and positively related (Fig. 2) with slope β = 1.736 (95% credible interval (CIs) 1.466–2.058) confirming that fractionation of δ^34^S in the hair reflects the δ^34^S in the surrounding grass [7, 12]. Furthermore, the intercept (i.e. the baseline fractionation rate between grass to herbivore tissue) was − 4.670 (95% CIs − 7.563 - -2.279) coincident with the estimated range of fractionation rates from previous studies [12, 18]. With segment-level standard deviation of *σ*_*ε*_ = 0.64, and individual level standard deviation of *σ*_*α*_ = 0.292, segment level variation in δ^34^S_*T*_ accounted for 83% of overall variation in recorded δ^34^S_*T*_ values.

### Power analysis result

**Fig. 3:**
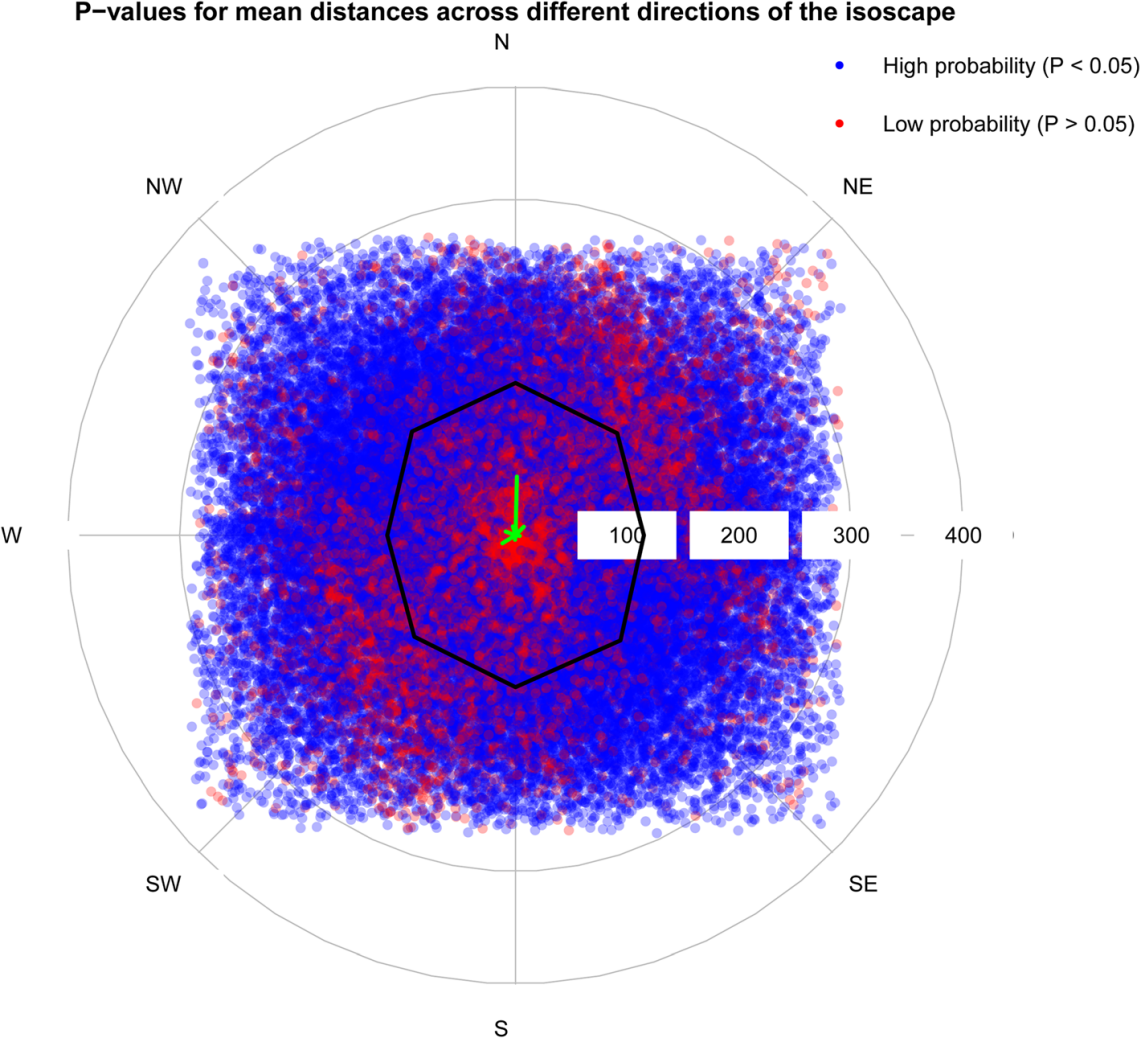
Black polygon shows mean distance required to move to detect movement across the Serengeti isoscape in different directions; green lines show the actual distance travelled by our cattle; red points correspond to distances and directions in which sulfur values in tail hair are not predicted to change significantly (based on comparing two segments, *P* > 0.05), while blue points correspond to distances and directions in which movement is predicted to result in a statistically significant change in sulfur tail values (*P* < 0.05)

The standard deviation on predicting tail hair δ^34^S values from the isoscape was typically very close to 1 (range 0.987–1.184). Our power analysis results suggested that an animal needed to move typically about 100 km (Fig. 3) in order to generate a difference in tail hair δ^34^S values likely to be judged significant. However, the nature of our sulfur isoclines across the Serengeti isoscape suggests effective distance is not equal in all cardinal directions. For instance, animals moving in a north-east to south-west pattern would in general have to move less than animals moving due north or south or east-west for the movement to be reliably detectable using δ^34^S (Fig. 3). Additionally, a questionnaire report suggested that our sampled cattle had not moved enough distance to establish their movement pattern using sulfur stable isotopes ratios, because the highest distance travelled by the cows was 52 Km with the majority having travelled less than 10 Km (See Additional file 1; Appendix 4).

## Discussion

The application of sulfur isotope ratios to quantify the movement of animals delivers considerable benefits. The major findings from our study suggest that: 1) sulfur isotope ratios in the vegetation across our study area vary across lithology; 2) sulfur isotope ratios in the vegetation are accurately reflected in cattle tail hairs (Fig. 2), and therefore 3) variation in tail hair could be used to potentially identify non-local movements of animals.

Feeding studies for grazing animals have derived fractionation factors of − 1‰ [12] and 1.2 ± 0.3 ‰ [18] between fodder and animal keratin. This is a relatively small change when compared with the range of values we have from grass samples across the Serengeti ecosystem and indicates the potential of sulfur isotope values as a dietary tracer within this system. The mean distance required to detect movement is about 100 km and modest compared to how far cattle or other animal species can move across this system and beyond. For example, pastoralist cattle can be transported by lorry many hundreds of kilometers across the country, and wildebeest which undertake a routinely cyclic migratory pattern every year can move about 250 km from southern to northern part of the ecosystem. The effectiveness of δ^34^S in studying movements for animals moving less than this distance in the Serengeti ecosystem would be dependent on local isotopic gradients.

### Variation of sulfur stable isotope ratios across the Serengeti ecosystem

The major source of sulfur in terrestrial plants is from soil, which is mostly derived from bedrock weathering in the form of sulphate [52]. In addition, the δ^34^S of soil is influenced by local lithology and rainfall [13, 51], aerobic and anaerobic growing conditions [5], microbial processes [13, 16], fertilization procedures, as well as atmospheric deposition including the sea-spray effect [53]. Normally, the δ^34^S value for bedrock varies with rock type and age [16, 54], and terrestrial plants exhibit a wide range of δ^34^S values between ∼ − 10 and + 35 ‰ [18, 53, 55]. In the Serengeti ecosystem, grass samples have δ^34^S values of 2.82‰ to + 13.04‰ (See Additional file 1; Appendix 3), midway in the global range. The lowest δ^34^S values in the Serengeti were observed on the eastern side of the ecosystem (Fig. 1b), which is dominated by volcanic ash tephra. The highest δ^34^S values in the isoscape come from the extreme south-eastern side of the Serengeti ecosystem beyond the Ngorongoro rain shadow (Fig. 1b) which is characterized by soils from pyroclastic-alkaline and volcanic lavas. The areas with the strongest gradients in the isoscape are south-eastern and north-western parts of the ecosystem (Fig. 1b), suggesting that animal movement across these isoclines could be detected over relatively short distances. In addition, the areas exhibit intermediate levels of mean annual precipitation of 800-1000 mm [56] (Fig. 1a) providing plants growing in the area with an important nutrient for their growth.

GAM results suggest the high values of sulfur in this region are mainly correlated with lithology and rainfall. However, microbial processes and volcanic activity in the Rift Valley [57] likely contribute to these high δ^34^S values. For example, the southern Serengeti plains are composed of tephra from volcanic eruptions from Ngorongoro highlands and other adjacent volcanoes including Lemagurut and Olmoti [58]. Volcanic gases and rocks have a wider range of δ^34^S values caused by inorganic chemical reactions, and this can change the δ^34^S signature of the soil [54, 59]. Aerosols and dust from volcanic fumaroles and eruptions can be dispersed by both prevailing winds and rainfall. The potential to include environmental variables in predicting sulfur variation, offers a useful predictive power to the isoscape, suggesting the method could be applied to other landscapes, particularly where there is soil heterogeneity and geological gradients.

The nature of the Serengeti sulfur isoscape suggests that sulfur isotopes ratios are relatively invariant across the centre and more heterogeneous across North East (NE) and South West (SW) gradients (Fig. 1b). Such a pattern indicates that an animal needs to travel longer distances along the East-West direction than NE-SW direction to detect movement (Fig. 3).

### Sulfur stable isotopes in ecological studies

This study has demonstrated the applicability of sulfur stable isotopes ratios in studying the movement of animals. Historically, sulfur stable isotopes have been difficult and expensive to analyse compared to carbon and nitrogen [16, 60, 61], which limited their applications to different studies [62]. The recent advances in continuous-flow isotope-ratio mass spectrometry (CF-IRMS) have enabled sulfur isotopes to be measured and analysed from both organic and inorganic materials in relatively small amounts [12, 63, 64]. This has increased their applicability in different fields of study including archaeology and ecology [12].

**Fig. 4:**
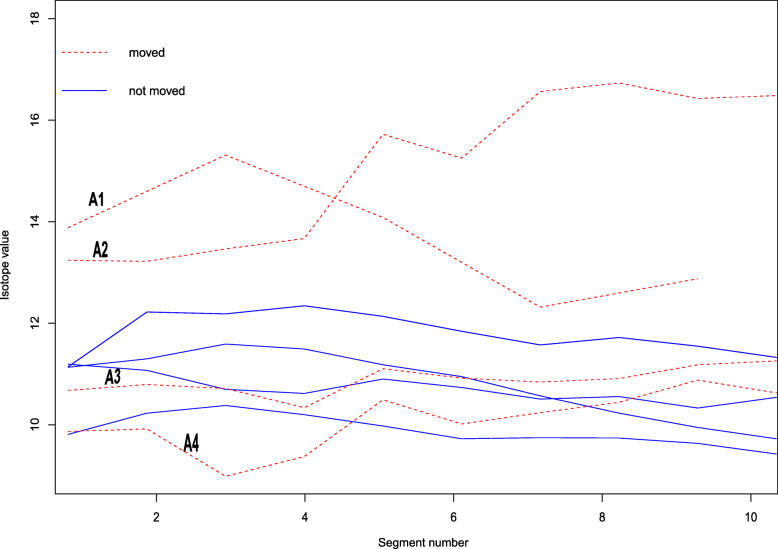
Variation of δ^34^S across length of tail hairs for representative individual cattle in Serengeti, showing sulfur isotopes profiles for reportedly moved (with their associated distance moved. A1 = 10.607 Kms, A2 = 13.636 Kms, A3 = 3.79 Kms and A4 = 9.318 Kms) and animals that did not move

Low variation in δ^34^S values of our cattle tail hairs (Fig. 4) suggests that cattle either foraged in a single location or foraged within an isotopically similar region of the isoscape (i.e. along the same isocline). Conversely, high variation in δ^34^S values in the tail hair (Fig. 4) should be taken as a primary indicator of movement or supplementary feeding using forage grown in a different isotopic setting [7]. This finding agrees with other recent works including that of Zazzo et al. [7], which revealed changes in δ^34^S in hair following the movement of sheep relative to the proximity to the sea.

### Estimating movement and wildlife home ranges of domestic and wild grazers

This study provides a template for understanding animal movement in and outside the Serengeti ecosystem. The question of where, how, and when livestock move remains critical for informing policies aimed at maximizing livestock productivity, minimizing disease spread while also maintaining traditional pastoralist livelihoods. Quantifying livestock movements, such as those occurring in remote agro-pastoralist settings, or through illegal transboundary trade or theft, is challenging to study using other methods. Our approach potentially provides a non-invasive way to infer whether an animal has moved long-distances across sulfur gradients in the previous ~5 months. The same methodology could be applied in other areas with sufficient variation in δ^34^S values where tail hair can be readily sampled (e.g. from carcasses) to study wide-ranging wildlife species (e.g. wildebeest and zebra). For example, this method could distinguish between migratory and non-migratory individuals and establish the frequency of different movement strategies in a partially migratory system such as Serengeti.

## Conclusions and recommendations

This study has shown the potential for using sulfur stable isotopes ratios in studying movement ecology of herbivores. For example, the δ^34^S isoscape of the Serengeti provides baseline information on how δ^34^S can be applied to understand spatial and movement ecology of livestock across the landscape. However, the use of a single element, such as δ^34^S does not capture all the isotopic variation across the landscape and could limit our ability to recreate detailed movement patterns of individual animals. The inclusion of additional isotope tracers such as strontium (^87^Sr/^86^Sr), which tends to have a well-defined geological distribution [65], or δ^2^H which tends to differ by watershed [6], could improve the technique by adding isotopic axes from which the location can be more accurately triangulated. Future studies could incorporate data from other tracking techniques, such as GPS telemetry or genetic markers, to further calibrate these isotopic techniques which would ultimately improve our ability to forensically determine the movement history of unmarked animals.

## Supplementary information

**Additional file 1 Appendix 1.** Questionnaires. List of questions asked to cattle owners for exploring movement history of cattle. **Appendix 2:** Summary statistics and model selection table. **Appendix 3:** Grass sulfur isotopes across the Serengeti ecosystem. Laboratory results for δ^34^S data used to interpolate the isoscape. **Appendix 4:** Distance in Km moved by cattle as per questionnaire report. Distance is the straight-line measure between two points.

## Data Availability

All data used in the development of this manuscript can be found here: 10.5525/gla.researchdata.1065.
