# Supplementary material for: Tracking animal movements using biomarkers in tail hairs: a novel approach for animal geolocating from sulfur isoscapes
Source: Mov Ecol. 2020 Sep 18;8:37. doi: 10.1186/s40462-020-00222-w (PMC7501629; doi:10.1186/s40462-020-00222-w)
Supplement: Supplementary file 1 — Additional file 1 Appendix 1. Questionnaires. List of questions asked to cattle owners for exploring movement history of cattle. Appendix 2: Summary statistics and model selection table. Appendix 3: Grass sulfur isotopes across the Serengeti ecosystem. Laboratory results for δ34S data used to interpolate the isoscape. Appendix 4: Distance in Km moved by cattle as per questionnaire report. Distance is the straight-line measure between two points. [file 40462_2020_222_MOESM1_ESM.pdf]

Appendix 1: List of questions asked to cattle owners for exploring movement history of cattle

PARTICIPANT / ANIMAL INFORMATION SHEET

A) BASIC INFORMATION

Date……………………. District……………………… Site……………………

Tribe…………... Animal's colour…………. Sex……… Age (or estimated) ……………

GPS readings: X………………… Y…………………… Sample ID……………….

B. SPECIFIC INFORMATION

1. (a) Did you buy your animal?

   (b) If yes, where and when?

   (c) If not, where did you get it?

2. For how long have you been keeping this animal?

3. Where do you normally graze your animal?

4. (a) Have you ever grazed this animal outside your normal grazing area?

   (b) If yes, where? And for how long? Can you remember the dates?

5. (a) Have you recently moved your animal to different place

   (b)If yes, where? Can you remember the dates?

6. Why did you move it?

7. When did you bring it back? And why?

8. If a need arises, can you allow us to re-sample your animal next time?

Appendix 2. Summary statistics and model selection.

	Model	df	logLik	AICc	delta	weight
m2	S_isotope ~ Mafic.volcanic_dist + Volcanic.lavas_dist + Volcanic.ashes_dist + Granitoids_dist + Pyroclastics.._dist + s(x_proj)+ s(y_proj) + ti(x_proj,y_proj)	16	-152.5	345.61	0	0.999
m1	S_isotope ~ MAP + elev + soil_exchbases + Mafic.volcanic_dist + Granitoids..mi_dist + Sandy..gravell_dist + Volcanic.lavas_dist + Volcanic.ashes_dist + Paragneisses.._dist + Granitoids_dist + Fine.coarse.cl_dist + Pyroclastics.._dist + s(x_proj)+ s(y_proj) + ti(x_proj,y_proj)	22	-152.3	360.21	14.599	0.001

Appendix 3: Laboratory results for grass sulfur isotopes across the Serengeti ecosystem showing $\delta^{34}S$ values

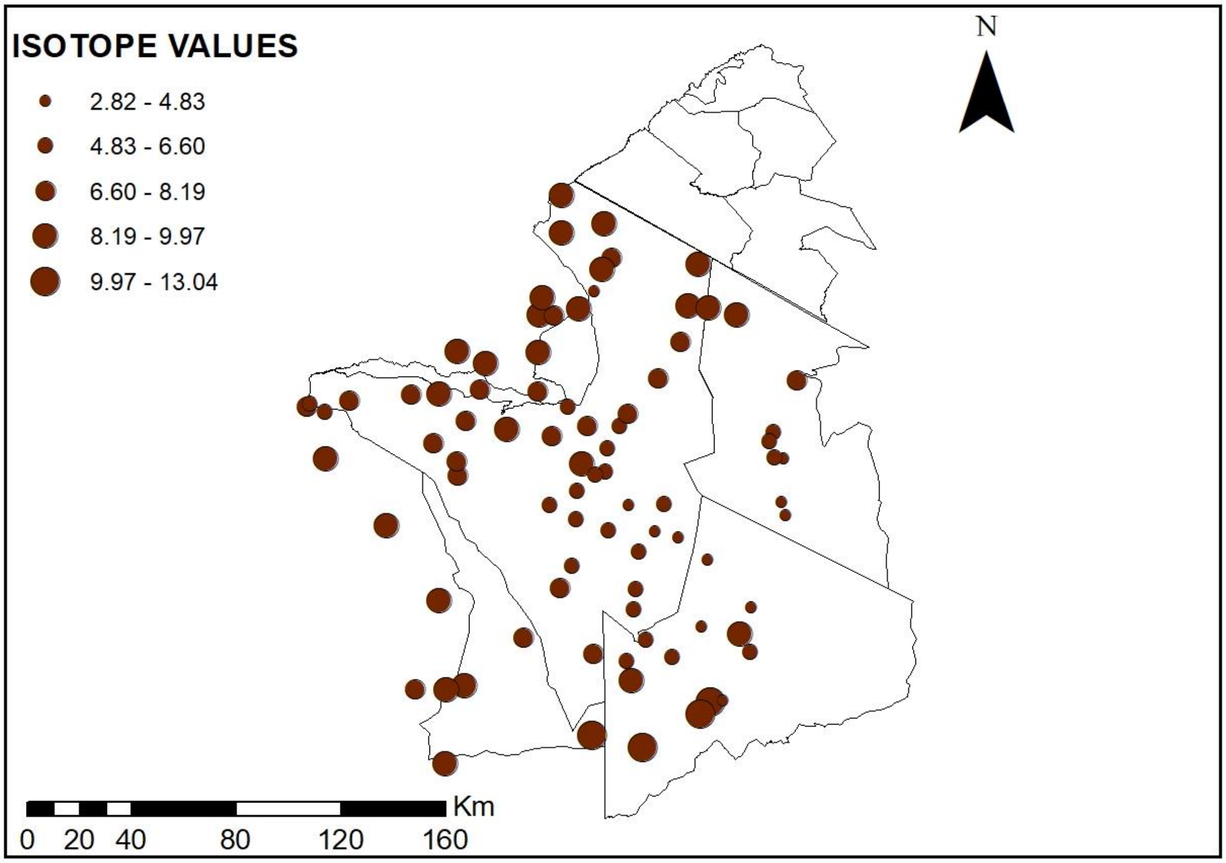

Appendix 4: Distance in Km moved by cattle as per questionnaire report. Distance is the straight-line measure between two points

ID	Location	Village_ sampled	X_coordinate	Y_coordinate	Date_moved	Sample_ collected	Village_ moved	X_coordinate	Y_coordinate	Movement Period	Cardinal direction (radians)	Distance Travelled (Kms)
A5	WESTERN	Robanda	686907	9763049	11/05/2017	05/08/2017	Machochwe	688141	9815346	2.9 Months	0.023591622	52.31155671
A7	WESTERN	Bwitengi	684832	9790956	23/04/2107	05/08/2017	Miseke	688575	9790360	3 Months	1.728701269	3.790153691
A13	WESTERN	Bonchugu	699163	9792946	20/03/2017	06/08/2017	Mugumu	690458	9796270	5 Months	-1.206034169	9.318047059
A22	WESTERN	Isenye	648172	9781707	Feb, 2017	06/08/2017	Iharara	647788	9777718	5 Months	-3.045623642	4.007440205
A24	WESTERN	Isenye	648413	9781854	June, 2017	06/08/2017	Iharara	9777718	647788	2 Months	-0.7851375	12.91415
A25	WESTERN	Isenye	648413	9781854	August, 2017	06/08/2017	Iharara	9777718	647788	Few Days	-0.7851375	12.91415
A27	WESTERN	Sapa	660020	9618602	March, 2017	08/08/2017	Ilindwa	656240	9616487	5 Months	-2.080922067	4.33146915
A28	WESTERN	Sapa	659839	9618378	March, 2017	08/08/2017	Mwanhuzi	648212	9611252	5 Months	-2.120635392	13.63697199
A30	WESTERN	Sakssaka	652669	9650201	July, 2017	09/08/2017	Butuli	653221	9656138	1 Month	0.092709718	5.962606225
A59	EASTERN	Endulen	752635	9644142	July, 2017	02/10/2017	Olepesi	753903	9647217	3 Months	0.391113985	3.326176333
A64	EASTERN	Esere	746519	9639601	August, 2017	02/10/2017	Endulen	753903	9647217	2 Months	0.76993273	10.60787029
A65	EASTERN	Esere	756247	9639293	August, 2017	03/10/2017	Endulen	753903	9647217	2 Months	-0.28760852	8.263420115
